# Periprocedural Considerations During Implantation of ICD In A Patient With Dextrocardia

**Published:** 2010-01-07

**Authors:** Farid Aliyev, Cengizhan Turkoglu, Isil Uzunhasan, Cengiz Celiker

**Affiliations:** Istanbul University, Institute of Cardiology, Department of Cardiology, Division of Pacing and Electrophysiology, Haseki-Fatih, Istanbul, Turkey

**Keywords:** ICD implantation, dextrocardia

## Abstract

In this case report we present a patient with dextrocardia, who undergone implantation of dual chamber implantable cardioverter-defibrillator (ICD). Here we aimed to underline several specific considerations which must be noted when one considers to implant an ICD in a patient with dextrocardia.

## Case Report

**Figure 1:**
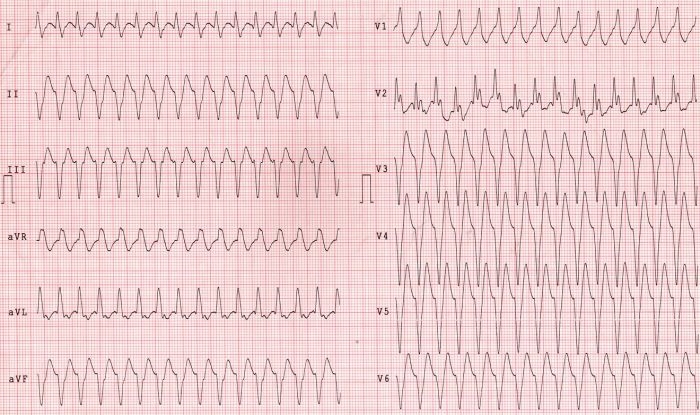
Episode of sustained ventricular tachycardia with haemodynamic instability. Precordial leads are placed on the right side of chest. Righ sided extremity electrodes are placed on the left and left electrodes are placed on the right side.

**Figure 2:**
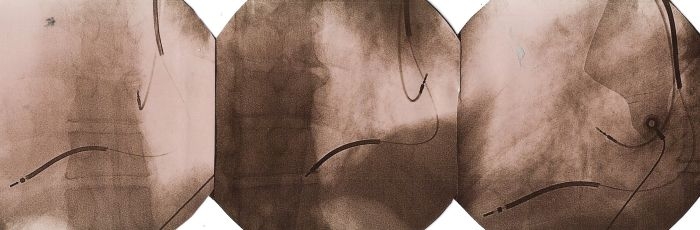
Fluoroscopic images obtained in anteroposterior view, 30º left anterior oblique view and 30º right anterior oblique view (from left to the right).

A 63 years old male with previously established diagnosis of dextrocardia, was admitted to our hospital with frequent syncopal episodes. He had documented episodes of sustained ventricular tachycardia (Figure 1) and normal coronary arteries. During electrophysiologic study, HV interval was measured 110 msec and fast ventricular tachycardia, degenerating to ventricular fibrillation was easily induced. We decided to perform implantation of dual chamber implantable cardioverter-defibrillator (ICD) (Figure 2). Procedure was successfully completed. Patient received 4 successful ICD discharges for ventricular fibrillation during one year follow-up.

## Discussion

Reports describing implantation of cardiac rhythm management devices in patients with dextrocardia were reported in the past. Most of these reports are dealing with implantation of permanent pacemakers and only small number present information about imlantation of an ICD. We suggest that implantation of ICD in patients with dextrocardia, have some differences from patients without dextrocardia. These are presented below:

1. Information about anatomy and/or coexisting congenital abnormalities, which may preclude percutaneous approach should be obtained before procedure.
2. Procedure must be performed with right-sided approach. Placing generator to the left side of the chest may result in high defibrillation thresholds or even insufficient defibrillation because of the fact that the critical myocardial mass lies on the left side. When we reviewed literature we found only three cases of ICD implantation in patients with dextrocardia. Two of them were performed with left sided approach [1,2] and one with right sided [3] approach. However authors who presented information about left sided approach, does not report any information about defibrillation threshold or patient outcome. Why should we use right sided approach in patients with dextrocardia. This question can be answered by asking another question - why we use left sided approach in patients without dextrocardia? Today we know that left ventricular mass is an important predictor of biphasic defibrillation threshold [4], and all new devices use defibrillator/pulse generator as active can which gives us an opportunity of changing of defibrillation polarity/vector. We also know that, implantation of ICD on the right side in patients without dextrocardia, frequently results in elevated defibrillation thresholds [5,6]. That is why it seems logical to implant an ICD in right pectoral region in patients with dextrocardia.
3. During right-sided approach, kinking of leads should be avoided, by creation of a wide generator pocket when compared to left sided approach. Current models of ICD's are designed according to implantation on the left side, so right sided implantation will result in kinking with subsequent risk of lead failure at its proximal site. In addition to creation of large pocket, this complication may be also prevented by implantation of generator upside down within the pocket.
4. And finally it will cause a quite different fluoroscopic appearance. Fluoroscopic images (Figure 2) are presented in anteroposterior view, 30º left anterior oblique view and 30º right anterior oblique view (from left to the right).

## References

[R1] Laborderie J (2007). Left-sided implantation of a biventricular implantable cardioverter-defibrillator in dextrocardia. Heart Rhythm.

[R2] Solanki KH (2007). Left-sided implantation of a biventricular implantable cardioverter-defibrillator in dextrocardia. Heart Rhythm.

[R3] Belotti G (2004). Biventricular cardiac defibrillator in dextrocardia with situs viscerum inversus. Ital Heart J.

[R4] Raitt MH (1995). Clinical predictors of the defibrillation threshold with the unipolar implantable defibrillation system. J Am Coll Cardiol.

[R5] Gold MR (2007). Low Energy Safety Study Investigators. Comparison of defibrillation efficacy and survival associated with right versus left pectoral placement for implantable defibrillators. Am J Cardiol.

[R6] Friedman PA (1999). Defibrillation thresholds are increased by right-sided implantation of totally transvenous implantable cardioverter defibrillators. Pacing Clin Electrophysiol.

